# Reply to: Regarding Iannicelli et al

**DOI:** 10.1186/s13052-021-01021-8

**Published:** 2021-03-28

**Authors:** Anna Maria Iannicelli, Daniele Vito, Concetta Anna Dodaro, Pasquale De Matteo, Rita Nocerino, Angela Sepe, Valeria Raia

**Affiliations:** 1 Department of Translational Medical Sciences, University of Naples “Federico II”, Via Pansini 5, 80131 Naples, Italy; 2 Department of Advanced Biomedical Sciences, University of Naples Federico II, Naples, Italy; 3 Department of Internal Medicine (Metabolic and Cardiac Rehabilitation Unit), University of Naples “Federico II”, Naples, Italy

**Reply**

Dear Dr. Chan and colleagues,

Thank you for your interest in our article, “*Does Virtual Reality reduce pain in pediatric patients? A systematic review*” (Ital J Pediatr. 2019 Dec 30;45 (1):171) [1].

While we absolutely agree with you on the need of clarification on a sentence in page 3 of the above mentioned manuscript, as you reasonably asked for, nevertheless we would like to expressly point out that an evident typing mistake has occurred which led to a frank misunderstanding. We accept responsibility related to this typo and apologize.

The correct sentence is “The sample size analyzed was numerically sufficient in the statistical analysis”, and not “The sample size analyzed was not numerically sufficient in the statistical analysis”.

You correctly highlighted that your group performed two studies (*n* = 123 and *n* = 131 respectively), both of which exceeded the a priori power calculation required of 114 patients per clinical setting [2]. Again, we absolutely agree with you also on this point. Actually, in Table 1 of our article we specified that the sample sizes of your studies are by far the largest in the literature.

We do appreciate your comment, which gave us the opportunity of a clarification.

Sincerely yours,

Anna Maria Iannicelli on behalf of all co-authors.
